# In vitro human cell culture models in a bench‐to‐bedside approach to epilepsy

**DOI:** 10.1002/epi4.12941

**Published:** 2024-04-18

**Authors:** Šárka Danačíková, Barbora Straka, Jan Daněk, Vladimír Kořínek, Jakub Otáhal

**Affiliations:** ^1^ Laboratory of Developmental Epileptology Institute of Physiology of the Czech Academy of Sciences Prague Czech Republic; ^2^ Department of Pathophysiology, Second Faculty of Medicine Charles University Prague Czech Republic; ^3^ Laboratory of Cell and Developmental Biology Institute of Molecular Genetics of the Czech Academy of Sciences Prague Czech Republic; ^4^ Department of Physiology, Faculty of Science Charles University Prague Czech Republic; ^5^ Neurogenetics Laboratory of the Department of Paediatric Neurology, Second Faculty of Medicine Charles University and Motol University Hospital, Full Member of the ERN EpiCARE Prague Czech Republic

**Keywords:** drug‐resistant epilepsy, genetic testing, in vitro human cell culture, legal and ethical aspects, precision medicine

## Abstract

Epilepsy is the most common chronic neurological disease, affecting nearly 1%–2% of the world's population. Current pharmacological treatment and regimen adjustments are aimed at controlling seizures; however, they are ineffective in one‐third of the patients. Although neuronal hyperexcitability was previously thought to be mainly due to ion channel alterations, current research has revealed other contributing molecular pathways, including processes involved in cellular signaling, energy metabolism, protein synthesis, axon guidance, inflammation, and others. Some forms of drug‐resistant epilepsy are caused by genetic defects that constitute potential targets for precision therapy. Although such approaches are increasingly important, they are still in the early stages of development. This review aims to provide a summary of practical aspects of the employment of in vitro human cell culture models in epilepsy diagnosis, treatment, and research. First, we briefly summarize the genetic testing that may result in the detection of candidate pathogenic variants in genes involved in epilepsy pathogenesis. Consequently, we review existing in vitro cell models, including induced pluripotent stem cells and differentiated neuronal cells, providing their specific properties, validity, and employment in research pipelines. We cover two methodological approaches. The first approach involves the utilization of somatic cells directly obtained from individual patients, while the second approach entails the utilization of characterized cell lines. The models are evaluated in terms of their research and clinical benefits, relevance to the in vivo conditions, legal and ethical aspects, time and cost demands, and available published data. Despite the methodological, temporal, and financial demands of the reviewed models they possess high potential to be used as robust systems in routine testing of pathogenicity of detected variants in the near future and provide a solid experimental background for personalized therapy of genetic epilepsies.

**Plain Language Summary:**

Epilepsy affects millions worldwide, but current treatments fail for many patients. Beyond traditional ion channel alterations, various genetic factors contribute to the disorder's complexity. This review explores how in vitro human cell models, either from patients or from cell lines, can aid in understanding epilepsy's genetic roots and developing personalized therapies. While these models require further investigation, they offer hope for improved diagnosis and treatment of genetic forms of epilepsy.

**Key points**

- Globally, epilepsy impacts 1%–2%, with one‐third resistant to conventional therapies.
- Research reveals diverse molecular pathways in hyperexcitability, extending beyond ion channels.
- In vitro human cell culture models promise robust pathogenicity and treatment testing.
- Model‐specific factors should be considered including clinical relevance, feasibility, time requirements, ethics, costs, and benefits.
- In vitro human cell culture models pave the way for personalized genetic epilepsy therapy.

## 1 INTRODUCTION

Epilepsy, one of the most frequent neurological disorders, can be caused by a variety of factors including brain injuries, stroke, infectious diseases, brain tumors, genetic conditions, malformations of cortical development, intoxications, etc., with approximately 30%–40% of cases having a genetic origin.^1^ Advances in genomic techniques during the past decade greatly extended our knowledge of the gene variations occurring across the entire human genome in many diseases including epilepsy. More than 1000 genes and their pathogenic variants have been identified to be associated with epilepsy, and this number keeps increasing steeply.^2^

Currently, systematic genetic testing is routinely conducted in both pediatric and adult patients presenting with epilepsy of unknown etiology, including those exhibiting malformations of cortical development (MCD) on magnetic resonance imaging (MRI). Biological samples such as blood,^3^ dysplastic brain tissue,^4^, ^5^ or, in rare cases, circulating DNA in the cerebrospinal fluid (CSF)^6^ serve as viable sources for DNA analysis, enabling the identification of germline variants (from blood) or somatic variants (from brain tissue or CSF). Methodologically, significant advancements have occurred in recent years, transitioning from single‐gene testing via Sanger sequencing to the era of next‐generation sequencing (NGS), encompassing targeted gene panel testing, whole‐exome sequencing (WES), and whole‐genome sequencing (WGS).^7^, ^8^

Disease‐causing variants in genes involved in epilepsy pathogenesis might affect various mechanisms and cellular processes. These mechanisms include signal transition, as in the case of channelopathies,^9^ energy metabolism (mitopathies),^10^, ^11^ or alterations in brain development, frequently caused by changes in the mTOR kinase signaling pathway (mTORopathies).^12^ Although specific gene variants have been identified in patients with epilepsy, their precise role in the process of epileptogenesis or ictogenesis usually remains unknown. By deepening our understanding of the contributions of particular gene variants to these processes, we can potentially enhance the treatment options and overall well‐being of individual patients. The diagnostic yield is highest (61.9%) in patients with seizure onset in their first month of life. In addition, genetic causes of epilepsy are detectable in 23% of adult patients with concurrent intellectual disabilities, potentially leading to improved well‐being for both patients and caregivers through changes in anti‐seizure medication (ASM).^13^

At the present day, a wide range of in vitro and in vivo methodologies are readily available, empowering researchers to investigate the effects of specific gene variants on various epileptogenic and ictogenic processes. High expectations surround these methods; however, they have many specific limitations including time demands or a restricted field of clinical relevance. In vitro methods using human cells seem to provide a reasonable model system to test relevant scientific as well as clinical hypotheses in specific cases. In this review, we will discuss the current and potential use of in vitro human cell culture models in epilepsy research, diagnosis, and treatment. We aim to address practical questions regarding their utilization and limitations, along with associated legal considerations.

## 2 IN VITRO HUMAN CELL CULTURE MODELS

**FIGURE 1:**
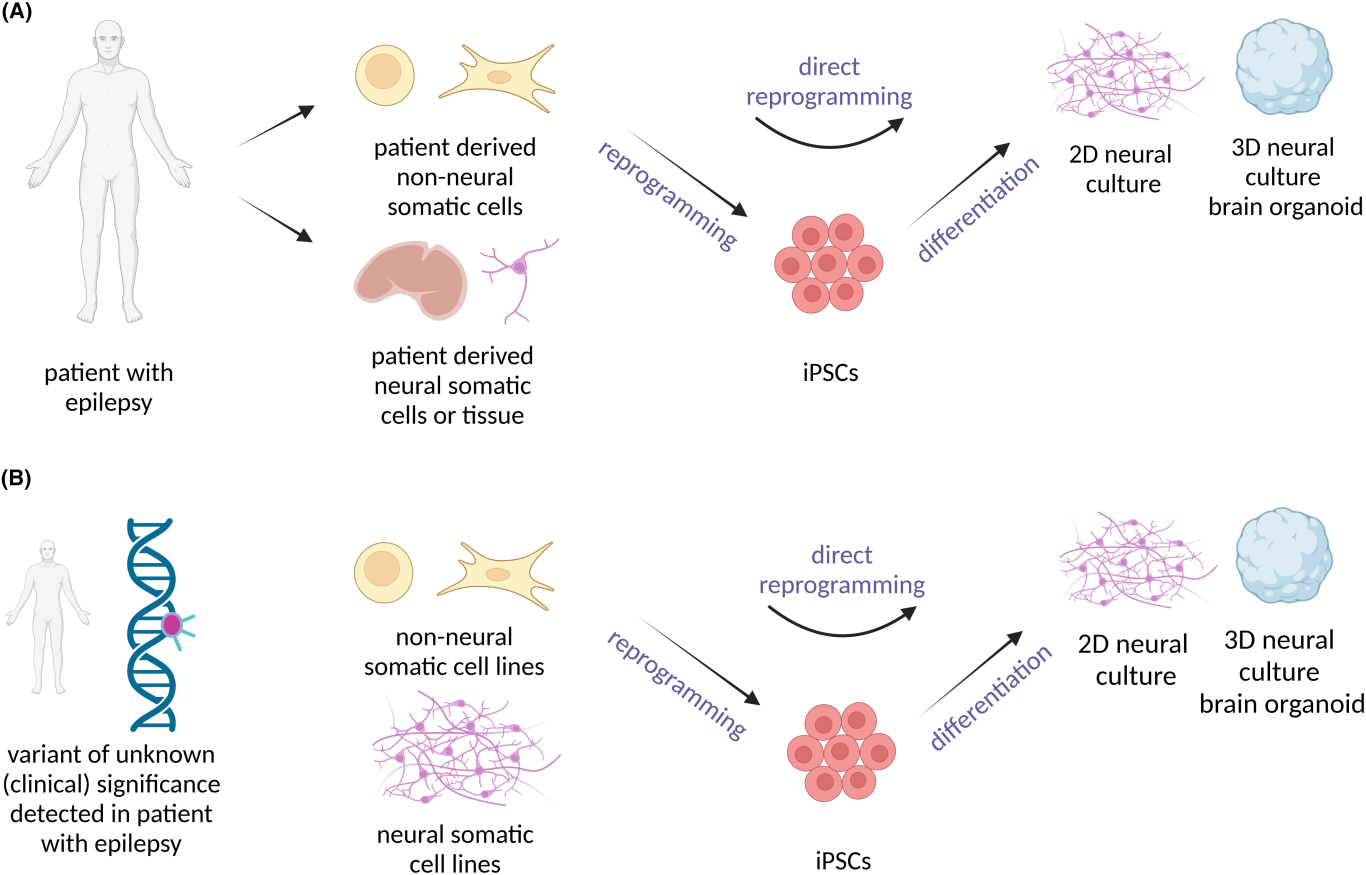
In vitro human cell culture models. (A) Patient derived cell culture models. Non neural or neural somatic cells can be acquired from patients with epilepsy. Non neural somatic cells (fibroblasts or blood cells) can be reprogrammed into induced pluripotent stem cells (iPSCs) and then further differentiate into two dimensional (2D) or three dimensional (3D) neural cultures. Another option is direct reprogramming of the non neural somatic cells into neural cultures without ever reaching the pluripotent stage. (B) Previously established cell lines. Another approach is testing the gene variants discovered during the gene screening of a patient with epilepsy. Detected gene variants can be introduced using various methods (transfection, transduction, genome editing using CRISPR/Cas9 or TALEN) into distinct characterized cell lines. Subsequently, the impact of the editing on the cell properties and function can be monitored. Created with BioRender.com.

Human cell cultures represent a crucial part of personalized epilepsy modeling both in terms of detecting the mechanisms of disease onset and development and selecting the appropriate therapeutic approach for the particular patient. Close cooperation between clinical centers and research laboratories is therefore increasingly emphasized. To properly select and implement a suitable model, it is necessary to understand their basic properties and limitations, including genetic stability, availability, maintenance requirements, scalability to allow high‐throughput screening, financial costs, established functional assays, etc. It is also important to consider how accurately the model mimics in vivo conditions and how it reproduces clinical manifestations and causes of epilepsy. At the cellular level, examination includes assessing the gene expression profile, the differentiation and maturation status, as well as the ability to manifest electrical or epileptiform activity. Another criterion for evaluating the affordability of the given model is whether the laboratory works reliably with the particular method and uses its technologies in a reproducible manner. We will primarily focus on two different approaches (Table 1). Firstly, we will discuss patient‐derived cells, both neural and nonneural somatic cells (and tissue), and their further processing (reprogramming, differentiation, or direct reprogramming) (Figure 1A). Second, the use of characterized cell lines, which can be manipulated to carry the specific gene variants detected during the genetic screening for epilepsy (Figure 1B). We will mention primary characteristics, benefits, and drawbacks as well as their use in translational epilepsy research. Selecting the most suitable model that would adequately represent a specific patient's condition will be vital in the future when disease models will be used to test and tailor individual treatment plans.

**TABLE 1:** Comparison of in vitro human cell culture models: patient derived cell cultures and characterized cell lines.

	Patient derived cell cultures	Characterized cell lines
Patient related strategy	Establishment of cell cultures for each individual patient	Cell lines with candidate variants applicable to multiple patients
Informed consent from patient	Cell culture establishment and maintenance	Genetic screening (identification of epilepsy related variant)
Cell model genetic background	Patient specific	Establish and well characterized
Cell model system development	Isolation of patient derived cells	Genetic manipulations of cell lines
Introduction of gene variants	No	Particular epilepsy related variants need to be introduced
Control samples	Isogenic cell line or non affected tissue	Parental cell line

### 2.1 Patient‐derived cell culture models

Cell cultures derived from particular patients are employed in a bed‐to‐bench approach to investigate genetic epilepsies. During the onset and development of epilepsy, the role of a single pathogenic variant within an epilepsy‐related gene is often complemented by the contributions of other gene variants to the disease phenotype and its diversity. In general, epilepsy should be assessed in the context of polygenic risk. This occurs even in cases of monogenic epilepsy, such as Dravet syndrome (associated with *SCN1A* variants), developmental epileptic encephalopathies (DEE), and other forms of epilepsy associated with intellectual disability.^14^, ^15^ For this reason, using cell models derived from the patient is highly advantageous due to the preservation of the genetic background. However, when using patient‐derived cultures, it is difficult to determine the contribution of individual gene variants to disease development. Thus, patient‐derived cells are being compared to isogenic controls, where gene variants detected in the patient have been repaired.

Modeling epilepsy involves the use of either nonneural cells (e.g., fibroblasts, peripheral blood cells) or brain tissue obtained during surgical resection of the brain.^16^, ^17^ Genetic screening is performed on these cells to determine the number of gene variants in epilepsy‐related genes. Patient‐derived neurons can be generated from nonneural cells, avoiding the need for surgical brain tissue resection. It is accomplished through reprogramming nonneural cells into induced pluripotent stem cells (iPSCs) with subsequent neuronal differentiation into two‐dimensional (2D) or three‐dimensional (3D) neural cultures^18^ or by employing direct reprogramming techniques.^19^, ^20^ In addition, patient‐derived cultures have application potential for selecting treatment based on the responses to drugs, without requiring knowledge of the patient's exact genetic background.

However, there are further aspects that should be considered. Utilization of patient‐derived cells is costly and time‐consuming since the cell culture must be individually and separately made for each patient. Moreover, the occurrence of the gene variants in the neural cells derived from nonneural somatic cells is not necessarily indicative of the presence of the same gene variants in the brain tissue. This is due to the possibility of brain‐specific mosaic variants (genetic alterations that are present in only a subset of cells within a brain). Then, we are not able to accurately model the in vivo condition.^21^ In addition, many international and national regulations need to be met when working with human cells, including their manipulation and storage, as well as their use in experimental research (see Section 2.3). We have compared in vitro patient‐derived cell culture models in terms of practical aspects related to culturing procedures (Table 2), as well as general and epilepsy‐related characteristics that are studied using these cultures, including appropriate methodologies (Table 3).

**TABLE 2:** Comparison of in vitro patient derived cell culture models: practical aspects related to culturing procedures (non neural cell cultures e.g. fibroblasts/blood cells, iPSCs derived 2D neural cell cultures,^22^ iPSCs derived 3D neural cell cultures,^22^ direct reprogrammed neural cell cultures,^23^, ^24^ resected human brain tissue cultures.^25^, ^26^, ^27^, ^28^

Patient‐derived cell cultures	Non neural cell cultures (fibroblasts/blood cells)	IPSCs derived 2D neural cell cultures	IPSCs derived 3D neural cell cultures	Direct reprogrammed neural cell cultures	Resected human brain tissue cultures
Basic characteristic	Represents pathophysiology of general cellular processes, and metabolism, suitable for genetic screening	Represents a broad range of neural cell types, providing a platform for disease modeling (synapses, electrical activity, neural proteins, neural networks, cell–cell interactions)	Models in vivo like conditions, allows complex interactions, stuctural changes, cell type composition, neurogenesis, cell migration, neural connectivity, electrical activity	Direct conversion to neural cells without going through the pluripotent state (synapses, electrical activity, neural proteins, basic properties of neural network, cell–cell interactions)	Reflects in vivo conditions, preserves cytoarchitecture, cell type composition, neural connectivity, electrical activity of epileptogenic neural tissue, suitable for genetic screening
Primary cells or tissue sample	Non neural cells	Non neural cells	Non neural cells	Non neural cells	Brain tissue
Presence of brain specific de novo somatic variants	Limited (depends on primary cells)	Limited (depends on primary cells)	Limited (depends on primary cells)	Limited (depends on primary cells)	Yes (brainarea dependent)
Invasiveness of cell or tissue collection	Non invasive (potentially invasive for obtaining skin fibroblasts)	Non invasive (potentially invasive for obtaining skin fibroblasts)	Non invasive (potentially invasive for obtaining skin fibroblasts)	Non invasive (potentially invasive for obtaining skin fibroblasts)	Invasive (brain surgery)
Developmental stage	Patient's age	Early stages of development	Early stages of development	Patient's age	Patient's age
*Demands for cell system establishment*					
Procedure	Easy	Challenging (requires iPSC generation from non neural cells and 2D neural differentiation)	Challenging (requires iPSC generation from non neural cells and 3D neural differentation)	Moderate	Challenging (requires surgical resection of brain tissue; from a specific subgroup of patients)
Time	Short (days–months)	Long (usually shorter than 3D cultures; 1–3 years) Ref. [22]	Long (1–3 years) Ref. [22]	Moderate (1–5 months) Refs. [23, 24]	Short (days–months) Refs. [25, 26, 27, 28]
Financial cost	Low	High (influenced by reprogramming and differentiation procedures)	High (influenced by reprogramming and longer differentiation procedures)	Moderate (influenced by direct reprogramming procedures)	Low (excluding the financial cost of the surgery)

Abbreviations: 2D, two dimensional; 3D, three dimensional; iPSCs, induced pluripotent stem cells.

**TABLE 3:** Comparison of general and epilepsy related parameters studied using in vitro patient derived cell culture models, including appropriate methodologies (non neural cell cultures e.g. fibroblasts/blood cells,^29^, ^30^, ^31^, ^32^, ^33^, ^34^ iPSCs derived 2D neural cell cultures,^35^, ^36^, ^37^, ^38^, ^39^, ^40^, ^41^, ^42^, ^43^, ^44^, ^45^, ^46^, ^47^, ^48^, ^49^ iPSCs derived 3D neural cell cultures,^32^, ^50^, ^51^, ^52^, ^53^, ^54^, ^55^, ^56^, ^57^, ^58^, ^59^, ^60^, ^61^ direct reprogrammed neural cell cultures,^44^, ^62^, ^63^, ^64^ resected human brain tissue cultures).^25^, ^26^, ^65^, ^66^, ^67^, ^68^, ^69^, ^70^, ^71^, ^72^

General characteristics	Epilepsy‐related characteristics	Non‐neural cell cultures (fibroblasts/blood cells)	IPSCs‐derived 2D neural cell cultures	IPSCs‐derived 3D neural cell cultures	Direct reprogrammed neural cell cultures	Resected human brain tissue cultures
Molecular and gene expression profiling		YES Refs. [29, 30, 31, 32, 34]	YES Refs. [36, 37, 38, 39, 40, 41, 42, 43, 44, 45, 46, 73]	YES Refs. [50, 51, 52, 53, 54, 55, 56, 57, 58, 59, 60, 61]	YES Refs. [44, 62, 63, 64]	YES Refs. [26, 67, 68, 69, 70, 71]
Detection of gene variants	Known or novel gene variants in epilepsy‐related genes	+	+	+	+	+
Variations in gene expression	Underexpression or overexpression of epilepsy‐related genes; neuronal excitability gene expression patterns specific for epilepsy; presence of specific proteins and ion channels (expression and function of ion channels associated with hyperexcitability)	+	+	+	+	+
Changes in cell signaling	Abnormal calcium signaling; activation and inhibion of the mTOR pathway	+	+	+	+	+
Available techniques		Next‐generation sequencing, Sanger sequencing, proteomics profiling, immunoblotting, qPCR, chemiluminescence	Next‐generation sequecing, Sanger sequencing, immunocytochemistry, immunoblotting, fluorescence microscopy, flow cytometry, qPCR, proteomics and transcriptional profiling, RNA‐Seq, ATAC‐Seq, calcium imaging, Sanger sequencing	Next‐generation sequecing, Sanger sequencing, immunohistochemistry, immunoblotting, fluorescence microscopy flow cytometry, qPCR, proteomics profiling, LC–MS/MS, RNA‐Seq, RNA, and DNA‐FISH	Next‐generation sequencing, immunocytochemistry, immunoblotting, fluorescence microscopy, flow cytometry	Next‐generation sequencing, immunohistochemistry, confocal microscopy, droplet‐based digital PCR
Morphology, proliferation, and cell type compositon		YES/NO Ref. [33]	YES Refs. [35, 36, 37, 39, 40, 41, 42]	YES Refs. [32, 51, 53, 54, 57, 58, 59, 61]	YES Refs. [62, 63, 64]	YES Refs. [26, 67, 68, 69, 70, 72]
Cell morphology	Soma size (dysmorphic neurons); spine density and morphology; dendritic overgrowth; synaptic markers; axonal sprouting	−	+	+	+	+
Cell proliferation and survival	Cell proliferation, abnormal neuronal and glial proliferation	+	+	+	+	+
The ratio of excitatory to inhibitory neurons	Increased excitatory activity; decreased inhibitory activity; altered exicatory/inhibitory balance in specific brain regions	−	+	+	+	+
Synapse and network formation	Abnormal synapse formation; synaptic markers; formation of aberrant neuronal circuits	−	+	+	+	+
Available techniques		Luminiscence, fluorescence	IFM, confocal microscopy, quantification of the dendrite bundles, axon initial segment imaging, neurite outgrowth assay, cell death assay, FRET, cell proliferation assay	IFM, confocal microscopy, synaptic puncta quantification, cell proliferation assay, morphometric analysis, radial glia‐like cells and heterogeneity analysis	IFM, confocal microscopy, morphometric analysis (areas, perimeters, and neurites features), proliferation assay, electron microscopy, apoptosis analysis	Immunohistochemistry, confocal microscopy, electron microscopy
Migration and development		NO	YES Refs. [41, 47]	YES Refs. [32, 57, 58, 59, 60, 61)]	YES/NO	YES Refs. [67, 68, 69, 70, 72]
Neuronal migration and development	Abnormal neuronal organization and connectivity (focal cortical dysplasia, lissencephaly, and heterotopia)	−	+	+	+/− (limited information)	+
Structural abnormalities	Gyral and sulcus patterns	−	−	+	−	+
Available techniques		−	IFM, cell migration assay, confocal microscopy, immunohistochemistry	IFM, confocal microscopy, neuronal migration assays, immunohistochemistry, qPCR	−	Widefield microscopy, immunohistochemistry
Energy metabolism		YES Refs. [29, 30, 31, 32, 33, 34]	YES Ref. [48]	YES Ref. [32]	YES Refs. [62, 63]	YES Ref. [71]
Mitochondrial function assessment	ATP levels; mitochondrial membrane potential; mitochondrial morphology; respiratory chain activity; reactive oxygen species production; and markers of mitochondrial biogenesis	+	+	+	+	+
Metabolic profiling	Increased glucose uptake and glycolytic activity; dysregulation of the TCA cycle; alterations in metabolite levels; impaired OXPHOS; neurotransmitter synthesis; alterations in lipid metabolism	+	+	+	+	+
Biomarker expression	Protein levels of mitochondrial enzymes; oxidative stress markers; glucose transporters; hexokinase; beta‐hydroxybutyrate; acetoacetate; glutamate/glutamine ratio; GABAergic biomarkers; lipid peroxidation products; metabolism of fatty acids	+	+	+	+	+
Available techniques		Spectrophotometry, respirometry, scintillation method, TEM, luminescence, IFM, flow cytometry, immunoblotting, biochemical techniques, qPCR, LC–MS/MS	Ceramide synthase assays, lipidomics	Enzymatic activity, thermal stability, and kinetic characterization, biochemical measurement	Mitochondrial membrane potential, network, and morphology, immunocytochemistry, determination of ROS, extracellular flux analysis, mitophagy analysis, flow cytometry	Spectrophotometric analysis, respirometry, mitochondrial translation assay
Electrophysiological properties		YES/NO Ref. [34]	YES Refs. [36, 37, 39, 40, 42, 43, 44, 45]	YES Refs. [51, 52, 54, 55, 61]	YES Refs. [44, 63, 64]	YES Refs. [25, 26, 65, 66, 72]
Cellular excitability	Hyperexcitability; changes in action potential firing rates; responses to external stimuli	−	+	+	+	+
Synaptic activity	Synaptic currents; neurotransmiter release; aberant synaptic activity	−	+	+	+	+
Neuronal networks activity	Disrupted synchronization of neuronal networks; aberrant network bursting activity; oscillations	−	+	+	+	+
Spontaneous electrical activity	Spontaneous neuronal firing; abnormal electrical activity; epileptiform discharges or bursts	−	+	+	+	+
Seizure‐like events (spontaneous or induced)	Ictal‐like discharges; paroxysmal depolarization shifts; spike‐and‐wave discharges; postictal depression	−	+	+	+	+
Response to drugs	Response to antiepileptic drugs and compounds targeting epileptogenic pathways	+	+	+	+	+
Available techniques		Respirometry	MEA, microarray analysis, optophysiology, intra/extra‐cellular recordings, ion selective electrodes, patch‐clamp, single‐cell electrophysiology, ATAC seq	MEA, optophysiology, intra/extra‐cellular recordings, ion selective electrodes, local field potential, patch‐clamp, cell‐attached recordings	MEA, microarray analysis, optophysiology, intra/extra‐cellular recordings, ion selective electrodes, patch‐clamp, single‐cell electrophysiology, ATAC seq	MEA, optophysiology, intra/extra‐cellular recordings, ion selective electrodes, local field potential, patch‐clamp, cell‐attached recordings

Abbreviations: 2D, two dimensional; 3D, three dimensional; ATAC Seq, assay for transposase accessible chromatin with high throughput sequencing; DNA FISH, DNA fluorescence in situ hybridization; FRET, fluorescence resonance energy transfer; IFM, immunofluorescence microscopy; iPSCs, induced pluripotent stem cells; LC–MS/MS, liquid chromatography tandem mass spectrometry; MEA, multi electrode array; OXPHOS, oxidative phosphorylation; qPCR, quantitative polymerase chain reaction; RNA‐Seq, RNA sequencing; ROS, reactive oxygen species; TCA, tricarboxylic acid cycle; TEM, transmission electron microscopy.

#### 2.1.1 Patient‐derived somatic nonneural cells

The most accessible method for studying genetic epilepsies using patient‐derived human cells is the employment of somatic nonneural cells (e.g. dermal fibroblasts, peripheral blood cells, buccal cells, exfoliated cells in the urine, and other cell types^74^, ^75^, ^76^). Even though of nonneural origin, these cells are commonly utilized for detecting pathogenic variants of epilepsy‐related genes^77^, ^78^, ^79^, ^80^ and as source cells for reprogramming into iPSCs.^21^ Furthermore, basic genomic and proteomic analyses, transcription profiling, biomarker analysis, drug screening, and metabolic analyses of the patient‐derived somatic nonneural cells are performed (Refs. [81, 82] for a review see Ref. [83]). The use of nonneural somatic cells has multiple advantages. First, the cells are easy to obtain from individual patients, enabling multiple sampling. Their cultivation process is straightforward, brief, and cost‐effective compared to other cell model systems. Second, nonneural somatic cells provide insight into the cellular pathophysiological processes of the individual case without the need to access the brain tissue from surgical resection.^17^ Finally, nonneural somatic cells can also be used for the first testing before more demanding methods, such as iPSCs generation (for a review, see Ref. [18]) or direct reprogramming (for a review see Ref. [84]).

Nonetheless, nonneural cells have their limitations. They do not represent the full diversity of cell types, cytoarchitecture, or biochemical environment found in the brain. An important drawback of nonneural cells is their lineage, since they may not correspond to the primary germ layer of the brain tissue – ectoderm. When exploring a specific gene or signaling pathway, it is advantageous to compare the gene expression levels in the analyzed cell type or tissue with the level in the brain tissue.^85^ Patient‐derived somatic nonneural cells are therefore used to study only a limited variety of cellular processes. When it comes to metabolic studies, we need to consider the difference between fibroblasts generating ATP based predominantly on glycolysis and adult neurons using mainly oxidative phosphorylation.^86^ Nonneural cells are not as widely utilized as iPSC‐derived cultures, except for genetic testing of gene variants.

Non neural somatic cells, mostly fibroblasts, have been successfully used to monitor changes in cell metabolism, cell signaling as well as responses to drugs. Metabolic alterations have been detected in fibroblasts derived from patients with mitochondrial diseases such as myoclonic epilepsy with the ragged red fibers (MERRF) syndrome^29^, ^30^ and mitochondrial encephalomyopathy, lactic acidosis and stroke‐like episodes (MELAS).^29^, ^31^ Cellular manifestations of MERRF and MELAS include decreased mitochondrial functions such as lower respiration rate, mitochondrial membrane potential, and decreased mitochondrial respiratory chain enzyme activities. Increased oxidative stress, mitochondria degradation, and abnormal calcium signaling have also been observed.^29^, ^30^, ^31^, ^87^ Fibroblasts derived from patients with developmental epileptic encephalopathy associated with a pathogenic variant in the UDP glucose 6 dehydrogenase (*UGDH*) gene have shown changes in UGDH stability, oligomerization, and enzymatic activity.^32^ Patient‐derived fibroblasts can also be used to test potential drugs. For example, one study has identified EPI 743 as a molecule useful for the prevention of ferroptosis, a form of regulated cell death characterized by the overload of intracellular iron ions. Therefore, EPI‐743 may be suitable for treating epilepsy‐associated mitochondrial diseases.^33^ In addition, it was found that ASM sulthiame, an inhibitor of carbonic anhydrase, selectively reduces mitochondrial function in fibroblasts derived from individuals with Leber's hereditary optic neuropathy (LHON) pathogenic gene variant. This effect contributes to the visual loss observed in patients treated with sulthiame.^34^ Although cell sampling is readily achievable, the utilization of nonneural somatic cells restricts the study to fundamental cellular pathophysiological mechanisms, such as cellular metabolism or drug responses, thereby limiting its applicability to a subset of epileptic syndromes.

#### 2.1.2 Patient‐derived induced pluripotent stem cells

Utilization of iPSC technology meant a breakthrough in disease research and modeling using in vitro human cell systems. iPSCs play a key role as an intermediate stage in the study of specific epileptic syndromes. The initial phase involves the reprogramming of nonneural cells (e.g. blood cells or fibroblasts) from patients into iPSCs. These iPSCs are then analyzed to confirm their pluripotency and undergo differentiation into 2D and 3D neural cultures (Figure 1A). In 2006, mouse fibroblasts were reprogrammed for the first time into iPSCs using a combination of four transcription factors (TFs) also called Yamanaka factors, namely Oct4, Sox2, cMyc, and Klf4, which were delivered by retroviral transduction.^88^ A year later, in 2007, the first human somatic cells (dermal fibroblasts) were reprogrammed into iPSCs using retroviral and lentiviral transduction.^89^, ^90^ Subsequently, the method became more prevalent and iPSCs were generated both from healthy individuals and patients with various diseases.^91^ Cellular reprogramming using nonintegrating vectors is a significant step forward in increasing the safety of the potential use of iPSCs in clinical practice.^92^ Different protocols for the differentiation of the iPSCs into numerous cell types were developed. At the same time, iPSCs have emerged as an alternative to human embryonic stem cell (hESC) lines, which are fraught with ethical controversies.^93^

A variety of somatic cells have been successfully reprogrammed into iPSCs: fibroblasts, peripheral blood mononuclear cells (PBMCs), urine‐derived stem cells (USCs), bone marrow‐derived mesenchymal stem cells, and other cell types. Notably, each cell type bears its distinctive features and inherent characteristics (for a review see Ref. [94]). In general, the ideal source cells should have the following properties: minimal or noninvasive cell sampling process, easy manipulation during the cell cultivation process, and high‐reprogramming efficiency at relatively low costs. These are mostly cell types that proliferate well under in vitro conditions and are capable of being frozen and stored at low temperatures (−80 to −196°C). Dermal fibroblasts are the most common cell type used for cell reprogramming. Fibroblasts are typically obtained by a punch skin biopsy, a simple procedure requiring the use of local anesthesia. In the next step, human biopsy samples are processed, and fibroblast cultures are established in vitro for further experiments. Fibroblasts are highly expandable in vitro and easy to manipulate.^91^ Although fibroblasts are commonly used as source cells with minimal‐invasive sampling, local anesthesia is still needed to minimize discomfort. Fibroblast cultures require an extended duration for establishment, ranging from 4 to 6 weeks, which substantially prolongs the overall timeline. The high expandability of fibroblasts can be a disadvantage if the fibroblasts overgrow the reprogrammed cells and simultaneously deplete growth factors in the medium.^95^ As a result, alternative shorter protocols relying on minimally or noninvasive procedures to sample source cells are being developed. PBMCs are easily collected from peripheral venous blood and isolated by density gradient centrifugation. Typically, about 10 mL of peripheral blood is needed from the patient for reprogramming, although there are protocols where less than 1 mL of blood is required. The immediate processing of PBMCs during the establishment of blood cultures is crucial to ensure their viability and functionality. Before reprogramming, cells are cultured in a medium enriched for cytokines such as SCF, FLT3, IL 3, IL 6, TPO, or IL 2 with anti‐CD3 antibody for 3–7 days.^96^, ^97^, ^98^, ^99^ In PBMCs, unlike fibroblasts, only reprogrammed cells adhere to the surface, while the other cells remain as a suspension in the medium.^99^, ^100^, ^101^ While not as commonly utilized for reprogramming, USCs possess unique attributes that make them an intriguing cell type. USCs are highly proliferative, multipotent cells with a high‐reprogramming success rate. Generally, about 100 mL of urine is needed for cell isolation and subsequent reprogramming. The collection of USCs from urine is completely noninvasive, unlike other types of somatic cells (Ref. [102]; for a review see Ref. [103]).

At present, diverse methods are employed to achieve cell reprogramming. One of the most common methods is retroviral or lentiviral TF transduction. However, this method possesses oncogenic risk due to the permanent integration of the viral genes into the genome.^89^, ^104^ A clinically safer approach is the use of nonintegrative methods such as the use of episomal vectors,^105^ Sendai viruses,^106^ modified mRNAs,^107^, ^108^ or microRNAs (miRNAs).^109^ The reprogramming process takes several weeks, approximately 3–4 weeks for episomal vectors when emerging colonies of iPSCs can be observed.^110^ Subsequently, they are manually picked and expanded, and their pluripotent state is confirmed. To confirm the pluripotency of reprogrammed cells, a series of experiments is required. These methods include assessment of colony morphology (round colonies, defined edges, dense center of colony, or high nucleus to cytoplasm ratio), chromosomal stability (normal karyotype), expression of pluripotent markers using RT–qPCR, or immunocytochemical staining of intracellular and extracellular pluripotent markers (SOX2, OCT4, NANOG, TRA‐1‐81, SSEA4). Other methods include the activity of alkaline phosphatase, DNA methylation assays, or embryoid body formation assay for assessing the ability of iPSCs to differentiate into all of the three primary germ layers.^99^, ^110^ A teratoma formation assay can also be performed by injecting the iPSCs into immunodeficient mice,^111^ though the usability of the assay is being discussed.^112^ The iPSCs can be cryopreserved at this stage and used for subsequent experiments after the successful completion of essential tests. IPSCs are further differentiated into either 2D or 3D neural cultures using different methods and protocols (see Sections 2.1.3 and 2.1.4).

The obtained iPSCs allow the generation of different cell types in vitro from an individual patient, enabling personalized modeling of a wide spectrum of diseases (cardiological, neurological, diabetes, etc.). They offer potential applications in regenerative medicine, although their implementation into clinical practice is currently hampered by their tumorigenic potential (for a review see Ref. [113]). Another potential use of iPSCs is a high‐throughput screening of candidate molecules (for a review see Ref. [114]). Disease models based on iPSCs are more commonly used for early‐onset diseases than for late‐onset diseases. One of the challenges for late‐onset disease modeling is an improvement in the maturation of iPSC‐derived cells. When generating patient derived neurons from somatic cells using iPSCs, the iPSCs are a stable intermediate state that can be confirmed and cryopreserved. This is unlike direct reprogramming, where the intermediate state is not usually well defined (see Section 2.1.5).^115^ It is challenging to establish a negative control when modeling diseases using patient‐derived cells. A negative control often comes from another healthy individual without a well‐characterized genetic background, which can affect the analysis. As a solution, isogenic controls have gained widespread usage. These controls involve the correction of the analyzed pathogenic gene variant to the reference wild‐type (WT) sequence within iPSCs. The acquired isogenic line preserves the patient's genetic background and allows for comparison between the mutated and WT cell lines. This enables assessment of the impact of the pathogenic variant itself. For example, when comparing an isogenic iPSC cell line acquired from a Dravet syndrome patient with a pathogenic variant in the *SCN1A* gene, increased expression of tyrosine hydroxylase and increased concentration of free dopamine in the culture medium in the patient's SCN1A cell line was confirmed.^116^

Cultivation of the iPSCs has several limitations. One of the main challenges is maintaining their pluripotent state. They require a stable environment and are sensitive to its changes. When conditions are not suitable, they spontaneously differentiate into other cell types. Researchers must be well‐trained to recognize the appropriate morphology and quality of iPSCs and to identify spontaneously differentiated cells. Once iPSCs are spontaneously differentiated, the parts need to be regularly removed, or in case of exceeding the limit of optimal quality, discarded and replaced with a new aliquot of iPSCs. To maintain pluripotency, growth factors are supplied in the culture medium. IPSCs grow on a special matrix (e.g., Matrigel, Geltrex, Vitronectin) or feeder layer (e.g., mouse embryonal fibroblasts). Antibiotics are often not supplied in the culture medium. Regular replacement of the culture medium is important, and it is often a daily routine. The entire process of iPSC cultivation is financially demanding and time‐consuming, often taking several months during reprogramming and confirmation of pluripotency. It is more convenient, but at the same time more expensive, to use commercial standardized cell culture media, since they have low variability of media composition. In addition, high variability exists between the iPSC clones derived from the same patient, so for a single experiment, it is necessary to include multiple clones and analyze each clone individually.^117^ The presence of variability among iPSC clones derived from different patients imposes additional challenges in terms of culture maintenance, particularly when compared to the use of well‐characterized iPSC lines known for their stability.

Despite possible complications with the generation and maintenance of iPSC cultures, they are an important part of epilepsy research today. Many iPSC lines have been developed, acquiring cells from patients with epilepsy or patients manifesting spontaneous seizures – Angelman syndrome, Rett syndrome, Fragile X chromosome, Phelan‐McDermid syndrome, *STXBP1* related epileptic encephalopathy, 15q11.2 microdeletion, tuberous sclerosis complex, Miller‐Dieker syndrome, Timothy syndrome, developmental and epileptic encephalopathy‐18 (DEE18) caused by pathogenic variants in *SZT2*, benign familial infantile epilepsy patient related to 16p11.2 deletion, Dravet syndrome, Unverricht‐Lundborg disease, epileptic encephalopathy with *CAD* deficiency, and other conditions (for a review see Refs. [22, 118, 119, 120, 121, 122, 123, 124]). The number of iPSC lines derived from such patients is constantly on the rise. Isogenic controls for iPSC lines derived from patients with epilepsy are also being developed to determine the impact of the specific pathogenic gene variant, as in the case of DEE caused by pathogenic variants in the *ARX* gene,^125^ Rett syndrome,^126^ Dravet syndrome,^116^, ^127^ tuberous sclerosis complex^35^ and others. In epileptic syndromes, where impairment to other types of tissues is often observed, patient‐derived iPSCs are utilized to generate not only neural but also other cell types, leading to a comprehensive understanding of the disease phenotype. While particular studies modeling epilepsy directly with undifferentiated iPSCs may be limited, research in this area could provide valuable insights into the early stages of epileptogenesis and help to identify potential targets for treatment. However, undifferentiated iPSCs lack the complex neuronal phenotypes seen in differentiated neural cells, which may limit their ability to fully recapitulate the epileptic phenotype. Therefore, most studies modeling epilepsy using iPSCs typically involve differentiation into neurons or other relevant cell types. In addition, several challenges related to reprogramming efficiency, generation of a sufficient quantity of reprogrammed cells, genetic stability, and standardization of protocols need to be addressed before iPSCs can be fully utilized in routine clinical practice. Further research is necessary to overcome these challenges and establish the clinical utility of iPSCs in epileptology.

#### 2.1.3 Patient‐derived 2D neural cultures

In vitro, 2D neural models, including the ones used in epilepsy research, are becoming increasingly popular due to the progress in iPSC technology. After the discovery of iPSCs, different protocols have been established allowing researchers to generate distinct types of neural cells such as: glutamatergic neurons,^128^ dopaminergic neurons,^129^ motor neurons,^130^, ^131^ or astrocytes.^132^, ^133^ Moreover, iPSCs are used to generate mixed cultures of neurons and glia in approximately 4–9 weeks.^134^, ^135^ The differentiation methods of patient‐derived iPSCs have been successfully standardized for a variety of cell types, for example, neural crest precursor cells derived from familial dysautonomia patients^136^ or neurons from patients with Rett syndrome.^137^

Protocols for the neurodifferentiation of iPSCs are mainly based on the application of small molecules or forced expression of TFs. Small molecules are being used preferentially in protocols for modeling physiological gene expression levels and neural cell development. Their substantial drawback is the time‐consuming procedure and the heterogeneity of the generated culture since every iPSC clone can display a variable response to the small molecules' exposure. Some of the small molecules being used include noggin, SB431542 as SMAD inhibitors, or LDN193189 as BMP‐mediated SMAD inhibitor.^138^, ^139^ During neurodifferentiation, the culture medium is often changed and enriched with other growth factors depending on the differentiation status.^140^ Protocols using TFs generally produce more homogenous neural cell cultures compared to protocols using small molecules, offering better standardization of differentiation procedures. The protocols employing TFs are less time‐consuming, but the conditional expression of additional TFs alters the physiological functions of certain signaling pathways. Commonly used TFs include neurogenin 2 (*NGN2*), which turns iPSCs into neurons within 14 days,^141^, ^142^, ^143^ or *ASCL1* and *DLX2*, which enable differentiation of iPSCs into GABAergic interneurons.^36^

The differentiation protocols further differ depending on the stages involved in the differentiation process: direct differentiation into neurons without the neural stem or progenitor cell (NSC/NPC) intermediate stage (around 2 weeks),^141^ or differentiation into neurons including NSC/NPC stage (1–6 weeks) (Ref. [144] for a review see Refs. [145, 146]). In the case of differentiation of iPSCs directly to post‐mitotic neurons, the neurons cannot be expanded further, must be used immediately, and the differentiation protocol has to be repeated from the beginning. The differentiation using NSCs/NPCs allows expansion and storage of the cells and does not require repetitive usage of iPSCs to reach differentiated neurons, making the process simpler. Fully differentiated neurons are suitable for electrophysiological in vitro experiments.^147^ Spontaneous electrical activity and action potential generation were detected in the culture of differentiated mature neurons and astrocytes 8–10 weeks after the differentiation onset (using iPSCs via NPC stage).^148^ Assessing neuronal maturation status during iPSC differentiation is an important characteristic, especially regarding inhibitory or excitatory activity. Upregulation of KCC2 expression during neural development is critical for the transition of GABAergic actions from excitatory to inhibitory. Impairments in KCC2 expression, associated with Rett syndrome, can be rescued by its overexpression, suggesting a potential therapeutic strategy.^37^ The ratio of excitatory glutamatergic neurons and inhibitory GABA interneurons should be considered when modeling seizure activity in mature neuronal cultures, as this ratio affects the culture's response to seizure inducing drugs.^149^ To model epilepsy, the protocols for the generation of glutamatergic excitatory cortical neurons,^140^, ^150^ GABAergic inhibitory interneurons (^73^, ^150^), hippocampal neurons,^151^, ^152^ glial cells,^153^, ^154^ or mixed neuron–glia cultures^134^ are thus particularly relevant.

The 2D monolayer neural cultures represent a suitable model for epilepsy research, although they have some limitations. Similar to the 3D cultures (see Section 2.1.4), 2D neural cultures exhibiting an immature phenotype are suitable for modeling early neural differentiation stages. Spontaneous electrical activity, neural networks, and formation of functional synapses have been observed in 2D cultures.^38^, ^155^ Compared to 3D cultures, 2D culture protocols are less time‐consuming and more amenable to manipulation when performing experiments. Their limitations include insufficient cytoarchitecture modeling and cell‐type composition, as well as the absence of an extracellular matrix, which is better represented in the 3D brain organoids. The generation of 2D (and 3D) neural cultures is preceded by work with iPSCs, which has further requirements (see Section 2.1.2).

Various types of genetic epilepsy and other neurodevelopmental diseases with seizures have been modeled using 2D neural cultures, including Dravet syndrome, PTEN macrocephaly, Miller‐Dieker syndrome, Rett syndrome, *STXBP1*‐related epileptic encephalopathy (for a review see Ref. [122]). Recent studies using GABAergic inhibitory interneurons derived from patients with *STXBP1*‐related encephalopathy have shown the presence of dysfunctional neural maturation as well as abnormal neural activity represented by reduced numbers of spontaneous spikes and bursts.^36^ IPSC‐derived hippocampal neurons from a patient with IQSEC2‐mediated disease have shown dysregulation of synaptic transmission as well as neuronal hyperexcitability.^39^ In general, 2D neural cultures exhibit epileptic phenotype like altered morphology (e.g. increased soma size and dendritic growth in TSC2‐deficient iPSC‐derived neurons,^40^ electrophysiological properties such as hyperexcitability of neural cells, generation of epileptiform activity after addition of convulsants or migration impairments).^41^ Patient‐derived neurons with epilepsy‐associated *SCN8A* variants were also utilized to test appropriate medication. Both phenytoin (a commonly used ASM) and riluzole (a drug used for amyotrophic lateral sclerosis) were successfully tested and led to a reduction in seizures.^42^ Another study employed patient‐derived neurons with the *SCN1A* variant, which were tested for common types of ASM.^43^

Patient‐derived neural cultures are valuable tool for testing both ASMs and off‐label drugs, facilitating the establishment of effective personalized treatment. Current efforts are aimed at achieving higher maturation, the appropriate ratio of excitatory to inhibitory neurons, and the number of glial cells. Also generating sufficient quantities of iPSCs and iPSC‐derived neural cells for personalized use and drug screening is necessary. There is a tendency for adding environmental factors to iPSC cultures (e.g., immunity, stress) to increase the validity of the results.

#### 2.1.4 Patient‐derived 3D neural cultures – Brain organoids

Brain organoids represent a self‐assembled 3D model that allows us to elucidate the characteristics of human brain development and model neurological diseases in vitro. Brain organoids, also called “mini‐brains”, are derived from the reprogrammed iPSCs, similar to 2D neural cultures. The first brain organoid was generated in 2013 by Lancaster and colleagues using iPSCs from healthy donors and patients with microcephaly.^156^ Subsequently, protocols have been developed for the generation of region‐specific organoids. These regions include the forebrain,^157^ hippocampus,^158^ midbrain,^159^ cortex,^160^ or cerebellum.^161^ The basic protocol for organoid generation is based on embryoid body formation using iPSCs or hESCs, followed by the induction of neural differentiation. Thereafter, the developing organoid is transferred into Martigel droplets to induce an extracellular environment. The organoid is then placed in a spinning bioreactor or orbital shaker, where an adequate supply of oxygen and nutrients is provided. Brain organoids can grow to a size of 3–4 mm in diameter. Further growth of the organoids leads to necrosis of its core due to the long diffusion distances resulting in insufficient nutrient and oxygen supply.^162^ To overcome this limitation, protocols for organoid vascularization are being developed. For example, the assembly of brain organoids with vascular spheroids or ectopic expression of the ETV2 variant of the human erythroblast transformation‐specific transcription factor (ETS) in human cortical organoids (Refs. [163] for a review see Ref. [164, 165]).

Brain organoids provide the opportunity to model structural changes and recapitulate the complex cytoarchitecture and cell‐type composition resembling human brain regions. They are also useful for studying neurogenesis, cell migration, neural connectivity, and interactions with the extracellular matrix. The brain organoid represents an excellent model of the early neural developmental stage of the human brain. Using single‐cell RNA sequencing, it has been discovered that the expression of genes contributing to cortical development (differentiation, migration, extracellular matrix development) is similar in brain organoids as in the fetal neocortex.^166^ Transcriptional profiles of forebrain organoids cultured for 3–8 weeks resemble fetal brains of 8–9 post‐conception week (PCW), while organoids cultured for 14 weeks resemble fetal brains of 17–24 PCW, in some regions up to 35 PCW.^157^ Brain organoids can be maintained in long term cultures for over 1 year.^167^ Brain organoids also exhibit electrical activity, which was first detected as the presence of slow neuronal calcium waves as well as action potentials resulting from electrical stimulation.^156^, ^160^ Neuronal oscillatory activity has been measured in cortical organoids cultured for several months, showing that glutamatergic and GABAergic signaling are essential for the generation and maintenance of these oscillations.^168^ Another study has confirmed that brain organoids are able to generate spontaneous neuronal activity after 8–9 months of cultivation, associated with the formation of dendritic spikes.^169^ For the study of cortical layer formation or electrophysiological measurements, it is possible to use organoid slices in an air–liquid interface culture, which enables a sufficient supply of oxygen and nutrients.^170^, ^171^

However, brain organoids also have considerable limitations. The main drawback of using brain organoids is their lack of suitability for modeling the postnatal and adult stages of brain development, limiting their use to the early to midgestational stage. A significant challenge is the extensive heterogeneity of brain organoids between the experiments; therefore, the effect of the gene variant must be quite clear to show significant results compared to the negative or healthy control. When differentiating patient‐derived iPSCs from 3D brain organoids, it is necessary to assess the extent to which the genetic background of the patient may influence the neurodifferentiation itself. Here, isogenic cell lines play an important role as an appropriate control. In addition, the excessive financial and time requirements of the experiment should be taken into account when considering brain organoids as a disease model for an individual patient.

Brain organoids have already been produced and used to study epilepsy and other neurodevelopmental disorders associated with seizures such as Angelman syndrome, tuberous sclerosis complex, Timothy syndrome, progressive myoclonic epilepsy type 1, and others (Ref. [50] for a review see Ref. [172]). Brain organoids derived from patients with Rett syndrome have demonstrated the capability of generating spontaneous epileptiform‐like activity in addition to abnormal neuronal oscillatory activity.^51^ Recent studies using epilepsy patient‐derived brain organoids have demonstrated an imbalance between glutamatergic and GABAergic neurons, cortical dysplasia, and enhanced astrogenesis in *WWOX*‐related epileptic encephalopathy (WOREE syndrome).^52^ Altered synaptic balance in the tuberous sclerosis complex has also been observed.^53^ Other recent discoveries include hyperexcitability, enhanced network connectivity, as well as downregulation of the small Ras homolog family member A (RHOA) GTPase in focal cortical dysplasia^54^; or neuronal hyperexcitability and ion channel dysfunction in *CDKL5* deficiency disorder (CDD).^55^ The Eichmüller group generated a human cerebral organoid model for tuberous sclerosis complex and identified a specific NSC type, caudal late interneuron progenitor (CLIP) cells. In the tuberous sclerosis complex, CLIP cells have been shown to proliferate excessively and generate enormous numbers of interneurons, brain tumors, and cortical malformations. In addition, inhibition of epidermal growth factor receptor (EGFR) significantly reduced the tumor burden.^56^

Brain organoids provide the possibility of thorough characterization of the processes behind the seizure development involving synaptic reorganization, abnormal migration, hyperexcitability, synchronized network activity, and even exhibit spontaneous seizure‐like events. While these characteristics may not fully replicate the complexity of seizures observed in vivo, they provide a valuable platform for research of the underlying mechanisms of epileptogenesis. However, further work is needed to make brain organoids clinically more relevant and applicable.

#### 2.1.5 Patient‐derived neural cultures generated by direct reprogramming

Direct reprogramming, also called transdifferentiation, directly converts somatic cells (in our context, patient‐derived somatic cells) into neural cells without reaching the pluripotent stem cell state.^173^ This approach represents another way to model neurological diseases and is also used in other fields, such as cardiology or diabetology (for a review see Refs. [174, 175]). The first direct reprogramming was performed by Vierbuchen, who reprogrammed mouse embryonic fibroblasts into neurons using TFs specific for a neural line: Acsl1, Brn2, Myt1l.^19^ The first direct reprogramming of human fibroblasts to neurons was performed using the same TFs (ACSL1, BRN2, MYT1L) with the addition of NEUROD1.^115^ Direct reprogramming allows the generation of post‐mitotic induced neurons (iNs) that cannot be further expanded, so the process must be repeated for each experiment.^176^, ^177^, ^178^, ^179^ Later, protocols were developed to generate induced NSCs and NPCs from fibroblasts, allowing differentiation into additional neural types, their expansion, and also cryopreservation of the reprogrammed cells.^180^, ^181^, ^182^ Fibroblasts (both human and animal) are currently the most common cell sources for direct reprogramming. Numerous protocols have been developed based on the use of TFs,^182^, ^183^ small molecules,^184^ mRNA,^185^ miRNAs,^186^ or their combinations.^23^, ^187^ When using TFs, it is important to consider not only the role of the TFs themselves but also their effect on chromatin remodeling, which could trigger the expression of silenced DNA or the silent expression of genes in the source cells.^188^

The reprogrammed cells do not reach the pluripotent state, which reduces the risk of tumorigenesis when transplanted to the host (patient) – a major benefit of direct reprogramming compared with the differentiation of iPSCs. This benefit unlocks the potential of directly reprogrammed neurons for clinical treatment, for example, in vivo reprogramming of neurons from astrocytes, with endogenous astrocytes as the source cells.^189^ Moreover, the reprogramming process is less time‐consuming and less costly compared to the process of generating iPSCs. Directly reprogrammed neurons also exhibit the same electrophysiological characteristics as functional neurons. NSCs can be observed within 30 days of culturing, and after 90 days they develop into differentiated neurons that show spontaneous postsynaptic currents, a negative membrane potential, or fast inward sodium and outward rectifying potassium currents.^23^ Functional excitatory cortical neurons can be generated by direct reprogramming within 25–38 days. These neurons have the potential for synaptic integration into the adult human cortex.^24^ It should be considered that directly reprogrammed neurons retain the epigenetic signature, age‐related properties, or mitochondrial dysfunction of the donor cells, in contrast to iPSC‐derived neurons, where the epigenetic and age‐related signature is reset. Therefore, iNs are useful to model late‐onset diseases in which the (age‐related) properties of donor cells remain unchanged.^190^, ^191^, ^192^

Disadvantages of direct reprogramming include the difficulty of establishing standardized protocols, its low efficiency, and the high variability of clones because it is impossible to confirm their developmental stage, unlike iPSCs, where pluripotency is confirmed by a wide range of tests.^193^, ^194^ Another drawback may be the limited number of post‐mitotic neurons, as they cannot be further expanded if their number is insufficient. This problem can be solved by using protocols to produce induced NPCs and induced NSCs. Unlike iPSCs, early development cannot be monitored with the use of iPSCs, so this approach is not suitable for studying developmental aspects of the disease.^190^

In epilepsy research, the use of direct reprogramming is limited to studying mitochondrial diseases. Villanueva‐Paz and colleagues generated iNs from dermal fibroblasts derived from patients with the MERRF syndrome. These patients had a single mtDNA variant that caused decreased mitochondrial respiration rate and increased Parkin‐mediated mitophagy.^62^, ^63^ The low efficacy of the reprogramming process and the high heterogeneity of the resulting cultures limit the use of direct reprogramming in epilepsy research in comparison to iPSCs‐derived neuronal cultures.

#### 2.1.6 Human brain tissue obtained from epilepsy surgery

An alternative, more targeted approach to personalized care involves utilizing human brain tissue acquired during epilepsy surgery. Because each patient is different and has a unique genetic background, personalized characterization of brain tissue properties (altered morphology and aberrant electrophysiological activity) or drug testing on the tissue has the potential to bridge the translational gap between preclinical and clinical drug development and answer a variety of clinically relevant questions.^25^, ^65^, ^66^ On the contrary, specific surgical techniques may complicate the use of brain tissue for research purposes. In addition, only selected patients with epilepsy undergo resective epilepsy surgery (or brain biopsy), the goal of which is to cure intractable epileptic seizures.^195^ Traditionally, only patients with structural focal DRE were considered suitable candidates for epilepsy surgery, with DRE defined as the failure of two adequately selected and tolerated ASM to achieve permanent seizure freedom.^196^ Today, the spectrum of candidates for surgical intervention is expanding to prevent adverse effects on development and cognitive abilities, particularly in children. Tissue processing and subsequent analysis must occur immediately after surgery because of the limited viability of the brain tissue sample.^26^ Therefore, close collaboration between the surgical and research teams in the surgical theater is required. Immediately after surgical removal of the affected brain tissue, a tissue sample is placed in an ice‐cold medium^197^ and immediately transported to a laboratory for further processing and evaluation of its properties. The tissue can be used either in the form of acute brain slices or in the form of organotypic slice cultures. Under optimal conditions, acute human brain slices remain viable for up to 12–48 h, allowing the use of various experimental methods, including electrophysiology and optophysiology.^25^, ^27^ The resected tissue can also be turned into organotypic brain slice cultures. This procedure prolongs their viability to 14–30 days.^26^, ^28^ After the recovery period, various properties can be assessed, including electrophysiological properties, morphology, metabolism, genetics, genomics, optogenetics, proteomics, molecular biology, and others.

The use of patient‐specific brain tissue samples has obvious advantages. They preserve the patient's unique genetic and epigenetic background, as well as specific clinical features, such as ASM use, type of epilepsy, and developmental stage of the patient. Genetic testing of the brain tissue allows researchers to detect the presence (or absence) of brain‐specific gene variants that arise during brain development and are not detectable by genetic testing of blood samples.^21^ Unlike other in vitro methods, only resected brain tissue preserves the original cytoarchitecture and neuronal connectivity. Working with ex vivo resected brain tissue involves relatively low financial requirements, and results can be obtained in a rather short time interval. Despite surgical removal of the suspected epileptogenic zone, some patients still experience intractable seizures. Examination of the resected brain tissue following surgical procedures holds promise in identifying the causes of surgical failure and providing valuable guidance for optimizing pharmacological treatment strategies.^198^ However, the standardization of tissue assessment procedures poses a considerable challenge in this context. The collection of tissue samples at varying time intervals, along with inconsistencies in sample size and brain region, complicates the establishment of uniform protocols. Consequently, the unique nature of brain tissue samples collected from patients simultaneously presents both advantages and disadvantages. While it allows for tailored analysis and insights into individual cases, the lack of standardization hinders efforts to ensure consistency and comparability across different samples. The use of multiple ASMs, different epilepsy characteristics, and the lack of a control tissue prevent us from drawing generalized conclusions about specific pathophysiological mechanisms based on the analysis of patient samples alone. Some data can be compared with information from animal models, but even this approach has its limitations.^197^

Numerous studies have been performed using resected brain tissue from patients with epilepsy. Typically, the presence of potentially pathogenic brain‐specific mosaic variants is detected using genetic screening, histopathological characterization,^91^, ^199^, ^200^, ^201^, ^202^, ^203^, ^204^ and electrophysiological and biochemical analyses.^197^, ^205^ Acute brain slices and organotypic cultures allow observation of electrical activity during electrophysiological measurements.^206^ Brain slices can develop spontaneous ictal‐like activity even without the presence of proconvulsants. Nevertheless, proconvulsants or electrical stimulation are regularly used to induce ictal‐like activity in these slices.^27^, ^207^ Brain and tissue banks such as the European or Canadian Epilepsy Brain Bank store frozen (−80°C) or formaldehyde‐fixed, paraffin‐embedded samples of brain tissue from resective surgeries for further use.^208^

In conclusion, in vitro human brain cultures offer a unique platform to explore the molecular mechanisms of epilepsy, investigate potential therapeutic targets, and personalize treatment strategies for patients with epilepsy. However, since the tissue is harvested from epileptosurgery resecate it possesses limited benefits for donor patients.

### 2.2 Models based on characterized cell lines

Epilepsy is also studied in vitro using characterized cell lines. These cell lines allow researchers to introduce patient‐specific and potentially pathogenic gene variants and observe their role in epileptogenesis.^209^ Cell lines are suitable for exploring novel rare gene variants detected by genetic testing whose impact is not yet known,^210^ or to study the effects of known recurrent gene variants localized in mutational hotspots of genes involved in the pathogenesis of epilepsy.^211^ The use of characterized cell lines offers several advantages: better defined genetic background of the cells, and easy access to the cells, which can be obtained either from public repositories or directly from research laboratories.

Introduction of a specific gene variant into characterized cell cultures can be accomplished by transfection (transient protein production)^212^ or viral transduction, which generates permanent and stable expression of the gene of interest.^213^ These methods are commonly used in the study of ion channel structure and their electrophysiological properties.^214^, ^215^ It is also possible to assess the effects of specific gene variants on various cellular processes such as membrane transport and signal transduction cascades. Characterized cell lines are also useful for drug screening and other applications. Commonly used lines include human embryonic kidney cells (HEK293) or cervical cancer cells (HeLa).^216^, ^217^, ^218^ Studying the properties of ion channels on nonneuronal cells has a major benefit. Nonneural cells do not express many receptors or ion channels, thereby minimizing the potential impact of endogenous expression on observed results. On the contrary, the cell line‐based model does not mimic the physiological neural environment, so the properties of ion channels expressed in the nonneural cells may not match those in neural cells. This problem is further aggravated by the presence of genetic abnormalities in certain utilized cell lines (Ref. [219] for a review see Ref. [220]). Therefore, a model was needed that would allow the study of gene variants in cells that naturally express the target genes to a similar extent as the original cell type does in vivo. Currently, TALEN and CRISPR/Cas9 are gaining popularity as cellular genome editing techniques that allow the introduction of a specific gene variant into a single or both alleles of the target gene, enabling precise modeling at the level of the in vivo state.^221^, ^222^ These technologies have enabled advancement in the modeling of various pathological conditions, including epilepsy. Nonneural cells as well as iPSCs and hESC lines have been targeted with TALEN or CRISPR/Cas9 and subsequently further differentiated toward 2D or 3D neural cultures (for a review, see Refs. [223, 224, 225, 226]). The development of genome editing techniques has allowed researchers to study the impact of numerous epileptogenic variants on various cellular processes. Using this approach, Quarishi has demonstrated the effects of the *KCNT1* gene variant on the excitability of neurons derived from human iPSCs.^227^ In another study, genome editing techniques were used to generate an isogenic cell line to compare the properties of the original and repaired *SCN1A* variants on neural network activity in a differentiated neuronal culture containing both excitatory and inhibitory neurons.^228^ A comparative study by Pantazis has shown that not all iPSC lines display the same parameters such as genetic properties, genomic stability in the process of CRISPR/Cas9 gene editing, or differentiation potential toward distinct types of cells.^229^ The gene variant is often introduced into the WT iPSC line before being differentiated into the neural line.^230^ Despite the advantages of this approach, its translational potential is limited, because the development of the brain as well as the patient's condition may not be due to a single isolated gene variant but to the patient's whole genetic background. To partially balance this problem, it is possible to compare three cell lines with each other: the patient‐derived neurons from the iPSCs, the characterized iPSC cell line that has the studied gene variant introduced, and isogenic control – patient‐derived cells where the pathogenic gene variant is repaired. This allows us to isolate the impact of the genetic background and assess the effect of the studied gene variant both isolated and in the context of the patient's genome. In summary, characterized cell lines offer a controlled and reproducible platform for investigating specific cellular mechanisms underlying epilepsy. Their accessibility and manipulability make them invaluable tools for advancing our understanding of epilepsy pathophysiology and developing more effective treatments.

### 2.3 Selected legal and ethical aspects of the used experimental approaches

The field is regulated by a subset of international and national laws as well as regulatory authorities. Since regulations develop over time, compliance with the experimental design and its legal covering should be checked periodically. Informed consent of the patient with the biological sample collection and the extent of its future use for research and/or other purposes should be addressed. Informed consent must follow a full subset of international and national laws and be in agreement with the current version of the Declaration of Helsinki and its national derivatives.^231^ A special issue that has to be addressed, well explained, and understood by the patient is the utilization of the patient's cells in the future: both for research purposes and (optionally) for commercial use such as drug development and testing. The lack of explicit statements covering future usability including its extent and their full understanding by the patient or legal representative might avoid or significantly limit their use. Consider a hypothetical scenario where a scientific team aims to create a cell culture derived from the patient's cells, with the prospect of utilizing it for future commercial drug testing purposes. However, it is important to note that in the absence of explicit inclusion in the initial informed consent, the utilization of such patient‐derived cell cultures for commercial applications is prohibited. Acquiring an additional consent specifically granting permission for such usage can be challenging, if not unattainable, due to various practical and ethical considerations. Also, permission to transfer the material to other scientific labs around the world using special material transfer agreements (MTAs) should be present in the consent. There is no general template to be used; therefore, special care is needed from the very early stages of the project when considering possibilities of the use of human‐derived cells or cell lines, a form of protection of intellectual property, even in the stages where licensing is rather unclear and far remote. One of the common loopholes in informed consent is the use of highly complex expert terminology, avoiding a full understanding of the meaning of the content. While there may not be a universally binding recommendation, it is important to consider that in cases where disputes arise, the evaluation of the document may be conducted by a judge or jury consisting of individuals without a background in biological science.

Last but not least, intellectual property protection should be considered. Research teams worldwide share their knowledge and material today to accelerate scientific discoveries. However, although various materials can be easily obtained from specific repositories (AddGene.org, wicell.org, and many others) after signing the general‐purpose MTA, the MTA typically restricts the use of the material strictly for noncommercial academic research purposes. It may then happen that research that has led to the development of a specific epilepsy model will not be legally compliant for commercial drug testing. Therefore, one should carefully analyze the legal issues to fully comply with the planned purpose at the very beginning of the project.

## 3 CONCLUSIONS

In conclusion, this comprehensive review has synthesized the current theoretical and practical knowledge surrounding the utilization of in vitro human cell culture models in epilepsy research, especially when considering a bench‐to‐bedside approach. While in vitro methods may initially project an illusion of simplicity and feasibility in studying the role of potentially pathogenic gene variants in individual patients, a more nuanced reality emerges. We have provided an extensive overview of these methods and models, elucidating their distinct characteristics, specific requirements, potential drawbacks, validity, and notable applications within the realm of epilepsy research.

Despite the methodological challenges, time commitments, and financial considerations associated with in vitro human cell culture models, they hold tremendous potential as powerful research tools for routine pathogenicity assessment. With further development and refinement, these models can become integral components in the development of personalized therapies for genetic epilepsies, serving as a solid experimental foundation for precision medicine approaches. By harnessing the capabilities of these in vitro models, significant progress can also be made in expanding our understanding of the underlying mechanisms of epilepsy, facilitating the identification of therapeutic targets, and ultimately improving treatment outcomes and enhancing the quality of life for individuals affected by genetic epilepsies. Continued exploration and investment in this field will undoubtedly contribute to the advancement of precision medicine and the optimization of therapeutic strategies for epilepsy and related conditions.

## AUTHOR CONTRIBUTIONS

SD wrote the first draft of the manuscript. JO and BS wrote sections of the manuscript. SD, JO, BS, JD, and VK contributed to the manuscript revision and approved the submitted version.

## FUNDING INFORMATION

Supported by grants from Charles University no. 343421, Czech Science Foundation no. GA22 28265S, and from the Ministry of Health of the Czech Republic no. NV19‐04‐00369. Supported by project no. LX22NPO5107 from the Ministry of Education, Youth and Sports of the Czech Republic: Financed by EU – Next Generation EU.

## CONFLICT OF INTEREST STATEMENT

None of the authors has any conflict of interest to disclose.

## ETHICS STATEMENT

We confirm that we have read the Journal's position on issues involved in ethical publication and affirm that this report is consistent with those guidelines.

## Data Availability

The review article does not contain any original unpublished data.
